# Immune function as predictor of infectious complications and clinical outcome in patients undergoing solid organ transplantation (the ImmuneMo:SOT study): a prospective non-interventional observational trial

**DOI:** 10.1186/s12879-019-4207-9

**Published:** 2019-07-03

**Authors:** Camilla Heldbjerg Drabe, Søren Schwartz Sørensen, Allan Rasmussen, Michael Perch, Finn Gustafsson, Omid Rezahosseini, Jens D. Lundgren, Sisse Rye Ostrowski, Susanne Dam Nielsen

**Affiliations:** 1 Viro-immunology Research Unit, Department of Infectious Diseases, Rigshospitalet, University of Copenhagen, Copenhagen, Denmark; 2 Department of Nephrology, Rigshospitalet, University of Copenhagen, Copenhagen, Denmark; 3 Department of Surgical Gastroenterology and Transplantation, Rigshospitalet, University of Copenhagen, Copenhagen, Denmark; 4 Department of Cardiology, Section for Lung Transplantation, Rigshospitalet, University of Copenhagen, Copenhagen, Denmark; 5 Department of Cardiology, Rigshospitalet, University of Copenhagen, Copenhagen, Denmark; 6 Department of Infectious Diseases and CHIP, Centre of Excellence for Health, Immunity and Infections and PERSIMUNE, Centre of Excellence for Personalized Medicine of Infectious Complications in Immune Deficiency, Rigshospitalet, University of Copenhagen, Copenhagen, Denmark; 7 Department of Clinical Immunology, Rigshospitalet, University of Copenhagen, Copenhagen, Denmark

**Keywords:** Immunomodulation, Organ transplantation, Immunosuppression, Infection, Graft rejection, Precision medicine

## Abstract

**Background:**

Solid organ transplantation (SOT) is a well-established and life-saving treatment for patients with end-stage organ failure. Organ rejection and infections are among the main complications to SOT and largely determines the clinical outcome. The correct level of immunosuppression is of major importance to prevent these complications. However, it is a consistent observation that in recipients on the same immunosuppressive regimens the clinical outcome varies, and no reliable marker exists to monitor immune function.

**Methods:**

In a prospective, observational study, we plan to enroll 630 adult patients with a planned organ transplantation at Rigshospitalet, University of Copenhagen, Denmark. Prior to and on different time points up to two years after transplantation we will perform a complete immunological profile on the recipients. This profile will consist of classical descriptive immune phenotyping (flow cytometry and circulating biomarkers) and the functional assay TruCulture®. In TruCulture® whole blood is incubated ex vivo with stimulants imitating bacterial, viral and fungal infections, where after a panel of selected cytokines is quantified. Clinical data from electronic health records will be obtained from the PERSIMUNE (Centre of Excellence for Personalized Medicine of Infections Complications in Immune Deficiency at Rigshospitalet, Copenhagen) data repository, a warehouse of data generated as part of routine care including vital signs, biochemistry, microbiology, pathology as well as medication, demographics, diagnoses, hospital contacts, surgical procedures and mortality.

**Discussion:**

This will be the first large scale study to determine several aspects of immune function and perform a complete immunological profiling in SOT recipients. It is expected that knowledge generated will provide information to generate prediction models identifying patients at increased risk of infection and/or rejection. If the study is successful, we will subsequently use the generated prediction models to propose personalized immunosuppressive regimens to be tested in future randomized controlled trials.

**Trial registration:**

This study has been approved by the Regional ethical committee (H-17024315), the Danish Data Protection Agency (RH-2016-47, RH-2015-04, I-Suite 03605) and the Danish National board of Health (3–3013-1060/1). The trial is retrospectively registered at clinicaltrials.gov (NCT03847285) the 20th February 2019.

## Background

Solid organ transplantation (SOT) is an well-established and life-saving treatment for patients with organ failure [1, 2]. Immunosuppressive drugs are required to prevent graft rejection but increases the risk of infections. Infectious complications are a leading cause of morbidity and mortality after SOT [3]. Pathogens and clinical presentations vary with type of transplanted organ, immunosuppressive regimens and infection-prophylaxis strategies. The most common infections are bacterial and viral, and the incidence of bacterial infections has been reported up to 30–68% per year in SOT recipients [4]. Acute rejection is reported in 27–46% of liver transplant recipients [5], 24% of kidney transplant recipients [6], 60% of lung transplant recipients [7] and in 56% of heart transplant recipients [8].

At present, immunosuppressive drugs are dosed according to weight and monitored by drug concentrations, and no reliable biomarker is available to guide dosing. In SOT recipients on the same immune suppression, some will develop severe infections or graft rejection, whereas others experience a good clinical outcome [1], indicating that the function of the immune system may contribute to the observed variation. Multiple studies have suggested that cytokines or specific cell populations may be biomarkers for SOT outcome, but so far no reliable marker has been identified (reviewed by Dendle et al. [9]).

The use of functional assays to determine immune function has gained attention as a possible tool to improve dosage of immunosuppression in SOT recipients [10–23]. Previously, functional assays have been expensive and required high level of technical skills and hands-on time in the laboratory, limiting the utility of this approach. However, recently commercially available functional assays such as the ImmuKnow®, QuantiFERON-monitor® and TruCulture® have become available, and large-scale functional assays are now feasible. Promising results were reported by Mian et al., who found that a low whole blood response of interferon gamma (INF-γ) after overnight stimulation with anti-CD3 (T-cell stimulant) and R848 (a Toll-like receptor 7 ligand), was associated with subsequent infections in SOT recipients [10], and Ravaioli et al. have found that immunosuppressive dosage according to results of ImmuKnow® improved the clinical outcome for liver transplant recipients [11].

There is an urgent need for improved understanding of the immunopathology contributing to risk of infections and rejections in SOT recipients. An improved understanding of the immune-pathophysiology combined with development of new immunologic diagnostic tools may promote a shift from empirical treatment to precision-guided care tailored to each patient, with expected improved patient outcomes.

In this “immune function as predictor of infectious complications and clinical outcome in patients undergoing solid organ transplantation (The ImmuneMo:SOT study)” study, we will examine SOT-recipients (prior to and after transplantation) with a complete immunologic profiling consisting of immune phenotype (high-dimensional flow cytometri), circulating biomarkers and the novel functional immune assay TruCulture®. The study is developed in collaboration with experts in infectious diseases, immunology and clinicians taking care of SOT recipients at Rigshospitalet, MATCH (Management of Post-Transplant Infections in Collaborating Hospitals, Rigshospitalet, Copenhagen, Denmark), PERSIMUNE (Centre of Excellence for Personalized Medicine of Infections Complications in Immune Deficiency at Rigshospitalet, Copenhagen, Denmark) and Milieu Intérieur, Institute Pasteur, Paris, France. It will be the first large scale study to determine multiple aspects of immune function and perform a complete immunological profiling in SOT recipients. We aim to 1) determine the combined effect of organ transplantation and immunosuppressive drugs on immune function, 2) determine if immune function profiling/monitoring can be used to predict infections and rejections, and 3) design a prediction a model to identify patients with the highest risk of infections. If the study is successful, these prediction models will be used to generate a treatment program using personalized medicine based on immune function that will be tested in randomized clinical trials.

## Methods/design

This ImmuneMo:SOT study is a non-interventional, observational study. It was initiated in February 2018 and inclusion will continue until March 2021.

### Study-participants

To be eligible for the study the participant must be a minimum of 18 years of age and have a planned kidney-, heart-, lung-, liver- or pancreas-transplant and be able to provide informed consent. Study participation is strictly voluntary.

During a three-year-period we aim to include 630 SOT recipients: *n* = 270 kidney-, *n* = 90 lung-, *n* = 45 heart-, *n* = 180 liver-, and n = 45 pancreas recipients from Rigshospitalet, University of Copenhagen [1]. A study nurse is employed to ensure adequate participant enrolment and complete follow-up.

### Complete immunological profile

In all participants we will collect blood samples before the transplantation (either while the patient is on waiting list or just before the surgical procedure and before the initiation of immunosuppression) and 7–14 days, 3 months, 6 months, 12 months and 24 months post-transplantation.

The complete immunologic profiling includes concomitant characterization of immune phenotypes and circulating plasma biomarkers and determination of immune function (Table 1). This immunologic profile is developed in collaboration with experts in infectious diseases, immunology and clinicians taking care of SOT recipients at Rigshospitalet, PERSIMUNE, MATCH and Milieu Intérieur, Institute Pasteur, Paris, France, the latter taking advantage of their experience with immunologic profiling in The Healthy Human Global Project [24–29].

**Table 1:** Method and data output of the immunological profile. Description of blood sample collection, laboratory analysis and data-output from the immunological profile, consisting of immune phenotype (flow cytometry), immune function (TruCulture®) and circulating plasma biomarkers

	Method	Data output
Immune phenotype (flow cytometry)	3 ml EDTA anticoagulated whole blood. The whole blood is analyzed on a Navios flow cytometer (Beckman-Coulter) within 24 h after blood sampling.	Proportion and intensity (mean fluorescence intensity, MFI) of antigens (most designated clusters of differentiation, CD) on the blood immune cells investigated in the flow cytometry panel.
Immune function (TruCulture®)	9 ml lithium heparin anticoagulated whole blood. The whole blood is transferred to individual TruCulture® tubes 60 min after blood sampling. After 22 h incubation at 37 °C, the TruCulture® supernatant is harvested and aliquoted to cryo tubes and frozen at − 20 °C for later thawing and bulk analysis of soluble immune activation products. The mRNA in the TruCulture® cell pellet is stabilized by Trizol and frozen at − 80 °C for later bulk analysis of mRNA expression level of stimulated immune proteins.	TNF-α, IL-1β, IL-6, IL-8/CXCL8, IL-10, IL-12p40, IL-17A, IFN-γ Expression levels of the immune protein mRNA included in the multiplex assay will be calculated and reported.
Circulating plasma biomarkers	6 ml EDTA and 3.5 ml sodium citrate 3.2% anticoagulated whole blood. The whole blood is spun (3000 RPM, 10 min) within maximum 6 h after blood sampling and plasma is aliquoted into cryo tubes and frozen at − 20 °C, and later transferred to − 80 °C. Plasma is thawed before analysis by Luminex®, MSD® or ELISA.	Circulating plasma levels of immune or inflammatory cytokines and autoantibodies against cytokines, chemokines, growth factors, adhesion molecules and injury/death products reflecting the magnitude of immune cell- and/or tissue activation and/or injury in vivo.

#### Immune phenotype

Immune phenotyping will be conducted in a subset of participants (*N* = 100–630 depending on funding). The flow cytometry panel will be designed to reveal the immune phenotypes of critical developmental and/or activation stages of immune cells to capture deviated maturation patterns, acute/chronic activation, exhaustion, migration/trafficking potential and deviated expression of immune checkpoint markers.

#### Circulating plasma biomarkers

Circulating levels of cytokines will be measured in a subset of participants (*N* = 100–630 depending on funding). The panel will include immune or inflammatory cytokines, autoantibodies against cytokines, chemokines, growth factors, adhesion molecules that injury/death products reflecting the magnitude of immune cell- and/or tissue activation and/or injury in vivo will be assessed by measuring the biomarkers. The soluble plasma biomarkers as well as autoantibodies against these will be measured by high-throughput multiplex platforms like Luminex® or Meso Scale Discovery (MSD®), by NanoString® and by enzyme linked immunosorbent assay (ELISA).

#### Immune function

To provide a standardized and robust analysis, immune function is assessed by the commercially available test TruCulture® (Myriad RBM, Austin, USA). TruCulture® reproducibly reveals the induced innate and adaptive immune response in whole blood after stimulation, by quantifying the release of soluble immune activation products (cytokines, chemokines, soluble receptors etc.) in the supernatant and by measuring the transcription level (mRNA) in the circulating blood (immune) cells [24, 30].

We have chosen four different stimuli, mimicking the presence of fungal, bacterial and two different viral agents, to obtain a broad function of different immunologic signaling pathways, including Toll Like Receptors (TLR) (Table 2). The four stimuli consists of heat killed *Candida albicans* (HKCA), bacterial endotoxin (lipopolysaccharide (LPS) from *Escherichia coli* (E.coli)), resiquimod R848 and polyinosinic:polycytodylic acid (poly I:C). Every tube holds one stimulus. HKCA is a whole microbe that provides a complex immunological stimulation including stimulation through TLR6. HKCA mimics the presence of a fungal infection. LPS-EB elicits a strong innate immune response trough TLR4, stimulating an antibacterial immune response. R848 is a synthetic agonist of TLR7 and TLR8 – both responding to single stranded RNA. PolyI:C is an analogue of double-stranded RNA, and activator of TLR3. Combined, R848 and PolyI:C, thus mimics the presence of viral infection. In addition to the stimulated tubes, one TruCulture® tube without stimulus serves as a negative control allowing for assessment of in vivo activation, which may be increased in some patients [24].

**Table 2:** Stimuli and immune response of TruCulture®

TruCulture® tube	Stimulus	Immune response
1	Heat killed *Candida albicans*	Whole microbe providing complex immune response, including activation of TLR6
2	Lipopolysaccharide from *E.coli* (LPS)	Bacterial endotoxin. Activator of TLR4.
3	Resiquimod R848	Synthetic agonist of TLR7 and TLR8 (both responding to single-stranded RNA)
4	Polyinosinic:polycytidylic acid (Poly I:C)	Analogue of double-stranded RNA. Activator of TLR3
5	None (negative control)	Allows for assessment of in vivo activation

Table 2 Showing the stimuli and response of the TruCulture® immune function test.

Whole blood is transferred to five different tubes. Four of the tubes are coated with stimulants, mimicking a fungal infection (heat killed *Candida albicans*), bacterial infection (LPS) and viral infection (Resiquimod R848 and Poly I:C). The fifth tube does not contain stimulant and serves as a negative control. This tube allows for assessment of in vivo activation. The whole blood is incubated for 22 h at 37 °C before the supernatant is harvested for analysis of biomarkers, the cells are stabilized with Trizol for analysis of mRNA. TLR: Toll-like-receptors.

Anticoagulated whole blood is transferred to individual TruCulture® tubes 60 min after blood sampling. After 22 h incubation at 37 °C, the TruCulture® supernatant is harvested, aliquoted, and stored at − 80 °C for later thawing and bulk analysis of soluble immune activation products (cytokines, chemokines, soluble receptors etc.) by Luminex®, Meso Scale Discovery® (MSD®) or ELISA.

The mRNA in the TruCulture® cell pellet is stabilized by Trizol and stored at − 80 °C for later bulk analysis of mRNA expression level of stimulated immune proteins. The technique applied for mRNA quality control and expression analysis will be commercially available and one that the laboratory is familiar with and applies at the time for mRNA expression analysis e.g. the multiplex assay nCounter GX Human Immunology Kit (NanoString Technologies) covering 511 human genes (shared among 24 immunology-related gene networks) known to be differentially expressed in immunology.

### Clinical data

Data from electronic health records will be obtained from the PERSIMUNE data repository [31]. These data are automatically generated prospectively as part of routine care and include vital signs, results of routine laboratory analyses of blood for hematology and biochemistry, microbiological examinations, results of imaging studies, pathological examinations of blood and tissue, medication, data on demographics, diagnoses, hospital contacts (outpatient visits and inpatient admissions), surgical procedures and mortality. The PERSIMUNE data warehouse collects data generated from routine patient treatment available for data extraction, as well as additional data from national registries and clinical databases. Patients are linked across data sources using their unique ten-digit civil registration number given to all Danish residents before pseudonymization. All data generated from this study will be stored at the PERSIMUNE data warehouse, approved by the Danish Data Protection Agency.

### End-points

Our primary endpoints are:

1. Infections within 1 year after the transplantation.
  - Blood steam infections
  - CMV infections
  - Pneumonia (virus, bacteria or fungi) requiring hospitalization
2. Graft rejection within 1 year after the transplantation.
  - Rejection defined by pathology
  - Definite or possible rejection that requires medical treatment

Secondary endpoints are:

1. Composite endpoint of infections (viral, bacterial or fungal) or graft rejection within 28 days, 90 days, and 2 years after transplantation

### Statistics and power calculation

#### Statistics

The data from the trial will be analyzed both separately for each patient category using classical statistical analyses and merged across the different patient categories and sampling time-points using computational modelling.

The data will be analyzed by classical statistical analyses i.e. descriptive statistics will be calculated for endpoints with summary statistics for continuous variables including n, means with standard deviation and medians with min/max or inter quartile ranges and summary statistics for categorical variables including n and proportions. Differences in continuous variables (including calculated delta-values) within groups between time-points will be analyzed by paired tests (t-test, Wilcoxon-signed rank test), mixed models or repeated measurements analysis (ANOVA, ANCOVA, Friedman), the latter followed by post hoc pairwise comparisons. Differences across different patient-groups or responses will be analyzed by two-sample t test or Mann-Whitney U test. Differences in categorical values will be analyzed by Chi-square tests or Fishers exact test as appropriate and McNemars test for changes over time.

The predictive value of immunologic variables or categorized immunologic variables for the endpoints will be analyzed by survival statistics including Kaplan-Meier plots and log rank test and Cox proportional hazards models. Furthermore, linear and logistic regression models will be applied to investigate the predictive value of immunologic variables for continuous and categorical endpoints. Analyses may be stratified by underlying disease/baseline patient characteristics, and odds ratios of outcomes among patients with an immunologic variable below or above a certain threshold will be estimated by logistic regression analyses adjusted for relevant covariates. The sensitivity, specificity, positive- and negative predictive value of specific immunologic variables or patterns for outcome will be calculated. *P*-values < 0.05 will be considered significant.

Furthermore, computational modelling of data applying a bioinformatics approach will be conducted by e.g. unsupervised learning to reveal patterns in the immunologic variables and profile predictive for the endpoints (data mining, clustering and/or Bayesian models, principal component analyses (PCA)).

#### Statistical power

The total number of patients planned investigated are *n* = 630 SOT recipients. We have performed power calculations to ensure adequate power:

#### Flow cytometry

Fernandez-Ruiz et al. [32] have found that a low NK-cell count (< 0.050 × 10^3^ cells/μL) at month one post liver-transplantation was associated with a greater risk of opportunistic infections at month one to six post transplantation. To detect such likely-hood ratio with α = 0.05 and power(β) = 0.80 for the incidence rate of 0,09 OI/1000 days vs. 0,58 OI/1000 days a total of *n* = 28 SOT recipients is required.

#### Plasma biomarkers

Several studies have reported that plasma sCD30 predicts infectious complications and acute rejection [33, 34] in kidney transplant recipients with a 64% vs. 75% 5-year graft survival in patients with high vs. low sCD30 levels [34]. To detect such likely-hood ratio with α = 0.05 and power(β) = 0.75, a total of *n* = 486 patients is required.

#### TruCulture®

To our knowledge, TruCulture® has not been used to investigate SOT recipients. In oncologic patients undergoing neoadjuvant radio- and/or chemotherapy before surgery, low IL-12 production in LPS stimulated whole blood cultures predicts increased sepsis-related mortality (58% vs. 6.6%) [35]. To detect such likely-hood ratio with α = 0.05 and power(β) = 0.80, a total of *n* = 24 patients is required.

## Discussion

The overall outcome of solid organ transplantation is largely defined by adverse events such as infections and rejections [4–8], and optimal dosage of immunosuppression is of utmost importance. Our general hypothesis is that an improved understanding of the immune function in SOT recipients will lead to an improved management of immunosuppressive therapy, fewer infections and rejections, and improved patient outcomes.

The ability to use functional assays to determine immune function to guide management of immunosuppression has been limited due to time-consuming and expensive assays. However, recently several commercially available functional assays have become available. The ImmuKnow® (Cylex, USA) is an FDA approved functional test for cellular immune function. The principle of the test is measuring intracellular adenosine triphosphate (ATP) production in CD4 + -cells upon whole blood stimulation with phytohemagglutinin (PHA) [15]. Although the technique is promising, many of the studies conducted with ImmuKnow® have limitations including retrospective design, small number of recipients, single measurement and/or low follow-up time, and results so far are conflicting [12–14, 16–23]. One promising randomized controlled study by Ravaioli et al. found that immunosuppression dosed according to results of serial testing with ImmuKnow® increased 1-year patient survival and lowered the incidence of infections in liver transplant patients [11]. Another currently available functional test of cellular immune function is the QuantiFERON®-monitor (QIAGEN). This assay is based on measuring INF-γ released after whole blood stimulation with innate (R848) and adaptive (CD3) stimulants [36]. Mian et al. have investigated 137 SOT recipients with the QuantiFERON®-monitor 1, 3 and 6 month post-transplantation and prospectively recorded infections. They found that INF-γ-levels were lower in patients that developed infections, and that a INF-γ-level < 10 IU/mL increased the likelihood of subsequent infection by 2- to 3-fold [10].

The TruCulture® is a novel functional immune assay that will provide complex information about the immune function [24]. TruCulture® was developed at the Institut Pasteur, France, with the purpose to be incorporated in the *Milieu Intérieur*-project, which is a large-scale study of 1000 healthy French adults [37]. In the ImmuneMo:SOT study the TruCulture® consists of four carefully chosen stimuli, mimicking fungal, bacterial and viral presence, acting through different TLR pathways. Furthermore, we have chosen 8 cytokines (TNF-α, IL-1β, IL-6, IL-8/CXCL8, IL-10, IL-12p40, IL-17A, IFN-γ) as readout providing a broad and representative image of the immune function. We expect this functional assay to provide much needed information about the global immune function of the SOT-recipients.

This ImmuneMo:SOT study is a non-interventional, observational, prospective study. The primary aim is to generate new knowledge about the immune function in SOT recipients and to link this information to risk of infections and rejections. When combining classical statistics and computational bio-informatic approaches to the data generated, it is expected that the study will be able to generate prediction models that can be used to design a treatment program using personalized medicine. Other ImmuneMo studies including other groups of patients undergoing immune modulating interventions are underway (patients treated with biological treatments, hematopoietic stem cell transplantation recipients, HIV-infected patients, patients with cancer, and patients with severe bacterial infections). Furthermore, a collaboration with Milieu Intérieur will provide the boundaries for the immune function in healthy individuals.

The major strength of this study is the complete immunological profiling that will be conducted in a large number of SOT recipients prior to and after transplantation and across organ types. The immunologic profile is developed in collaboration with the *Milieu Intérieur Consortium*, Institute Pasteur, Paris, France. This consortium initiated in 2012 a large cross-sectional healthy population-based study, to assess factors underlying immunological variance within the general healthy population. They have enrolled 1000 healthy Western-European adults; 500 women and 500 men, consisting of 100 study-participants of each sex in each of 5 age-groups from 20 to 29, 30–39, 40–49, 50–59 and 60–69 year. The study-participants was (among others) assessed with a deep immunological and genetic investigation, including the use of TruCulture® [25]. This provides us a unique opportunity to compare the immune function of our study-participants to healthy controls.

Another strength, and a precondition for the feasibility of this study, is that all Danish residents are provided with a unique 10-digit personal identification number by the The Civil Registration System. This number is registered at all contacts with the health care system and allows for accurate linkage with the clinical information from the PERSIMUNE data repository.

A weakness of the study is the single-center design. The patients recruited at one geographical locality and may not be representative for patients from other geographical areas. However, as Rigshospitalet is the largest center for transplantation in Denmark and the only center in Denmark for liver and lung transplantation, it does provide a unique opportunity to perform this study and it is realistic to include patients as described.

In conclusion, there is a need for an improved understanding of immune function in SOT recipients to target the constant challenge of balancing the immunosuppression to avoid both infections and rejections.

This project has brought together the competences of many experts, including expertise within infectious diseases, immunology, and transplantation medicine. We hypothesize that the full immunologic profile, consisting of both immune phenotyping, circulating biomarkers and immune function, will provide important knowledge about the effect of organ transplantation and immunosuppression on the recipients’ immune function. And that this knowledge can be used to identify SOT recipients at excess risk of infections and rejections.

If the study is successful, the study group will use the results to design a randomized clinical trial to test personalized immunosuppressive regimes according to the recipient’s individual immunological profile. In the future both initial immunosuppression and monitoring of immunosuppression might be based on the personal immune profile as proposed in this study.

## Data Availability

Use of data will be confined to the study group, but potential collaborators or request for data and/or other materials such as consent forms, information to participants and data-management documentation (in Danish) can be submitted at sdn@dadlnet.dk.

## Abbreviations

E.coli: *Escherichia coli*
ELISA: Enzyme linked immunosorbent assay
HKCA: Heat killed *Candida albicans*
LPS: Lipopolysaccharide
mRNA: Messenger RNA
MSD: Meso Scale Discovery
PERSIMUNE: Centre of Excellence for Personalised Medicine of Infectious Complications in Immune Deficiency
poly I:C: Polyinosinic:polycytodylic acid
SOT: Solid organ transplantation
TLR: Toll like receptors
